# The Importance of Person-Centered Approaches to Managing Behavioral and Psychological Symptoms of Dementia: A Case Report

**DOI:** 10.7759/cureus.37387

**Published:** 2023-04-10

**Authors:** Kayla S Murphy, David M Atkinson, Jamie Starks

**Affiliations:** 1 Psychiatry, University of Minnesota, Minneapolis, USA; 2 Psychiatry, The University of Texas (UT) Southwestern Medical Center, Dallas, USA; 3 Psychiatry/Geriatric Psychiatry, Minneapolis Veterans Affairs (VA) Health Care System, Minneapolis, USA; 4 Psychiatry/Geriatric Psychiatry, University of Minnesota, Minneapolis, USA; 5 Neurology/Behavioral Neurology, Minneapolis Veterans Affairs (VA) Health Care System, Minneapolis, USA; 6 Neurology/Behavioral Neurology, University of Minnesota, Minneapolis, USA

**Keywords:** veterans health, long term care, supportive and palliative care, behavioural and psychological symptoms of dementia (bpsd), cognitive impairment and dementia

## Abstract

Behavioral and psychological symptoms of dementia (BPSD) are common and associated with increased morbidity and mortality in dementia. In this report, we describe a patient with severe BPSD who was effectively managed with a variety of non-pharmacologic strategies. A 70-year-old Navy veteran and retired commercial flooring business owner with a history of dementia was admitted to the hospital with aggressive behavior. He was no longer manageable by his family. He required intermittent use of restraints and multiple antipsychotics during hospitalization. He spent much of his time crawling on the floor, “working” on floor tiles, which was often difficult for staff to safely accommodate. However, with time, interprofessional staff identified signs of distress and developed strategies to safely engage the patient’s current perception of his situation. This case highlights how BPSD may be driven by a person's identities and roles from earlier stages of life. Approaching and managing these symptoms flexibly can enhance dementia care.

## Introduction

Cognitive impairment is often regarded as the most prominent aspect of dementia; however, behavioral and psychological symptoms of dementia (BPSD) are also major features, occurring in up to 90% of individuals with dementia [1-2]. The most common of these symptoms include apathy, depression, irritability, agitation, and anxiety [3]. These symptoms are often a reflection of meaningful expressions of underlying distress. For example, apathy may signal disengagement, pacing a sign of discomfort, or physical aggression an indication of urgent need. If not addressed, these symptoms may escalate and negatively impact safety and care [4]. BPSD is also associated with reduced quality of life and increased morbidity and mortality [1]. Further, pharmacologic interventions commonly used for BPSD have at best modest evidence for efficacy, and many have significant risks and side effects. Therefore, recognizing and addressing BPSD and its underlying motivators is essential to person-centered dementia care. In this article, we present a patient with severe BPSD, often related to his former identity in commercial flooring, who was managed with a variety of non-pharmacologic management strategies from flexible and patient-centered staff.

## Case presentation

The patient first began experiencing symptoms of cognitive decline at age 62. His initial cognitive symptom was difficulty with arithmetic: trouble calculating tips at restaurants and managing finances, which his wife found puzzling given his background in accounting. He also had no history of mood disorders but began exhibiting irritability and anger. Before this, he functioned well for much of his life. He had been married for 45 years with two children, served in the Navy during the Vietnam War, completed four years of college, graduated with honors, and worked as an accountant, commercial flooring business owner, and most recently as maintenance staff for a school district.

Five years after the initial symptom onset, he developed difficulty completing tasks in physical therapy following a shoulder injury. This led to his initial cognitive workup. At that time, his Montreal Cognitive Assessment (MoCA) score was 11/30, and he was dependent on all instrumental activities of daily living (IADLs). Neuropsychological testing showed an amnestic pattern of deficits with severe cognitive impairment in nearly all domains. Fluorodeoxyglucose-positron emission tomography showed decreased uptake in the bilateral parietal and temporal lobes, with a slight decrease in the cingulate gyrus. Together, this suggests a diagnosis of early-onset Alzheimer’s disease.

Ultimately, at age 70, a crisis point occurred. He was still living at home, but he was dependent on others for activities of daily living (ADLs) and becoming increasingly aggressive toward his family. He was grabbing and hitting family members and could no longer be redirected or de-escalated. He was refusing medications and had not slept for 36 hours. Caregiver distress was severe, and living at home with his wife as a primary caregiver was no longer sustainable. The family brought him to the emergency department. Vitals, basic labs, and urinalysis were within normal limits. Given the patient’s clinical presentation and known history of dementia with behavioral disturbances, no further medical workup was pursued. Upon admission, he exhibited aggression, often striking staff during perineal care. He required frequent four-point restraints and 1:1 monitoring, making finding an appropriate long-term care facility difficult.

While in the acute medical wards for the next four months, he continued to strike out at staff, sing or yell loudly, and attempt to crawl on the floor, and thus physical restraints were intermittently used. When staff removed his restraints, he would often start crawling on the floor. He was a retired commercial flooring business owner, and it appeared he was "working" on floor tiles, often yelling at his employees and moving imaginary objects. He had severely impaired vision, likely secondary to cortical blindness, and aphasia characterized by word salad, which likely contributed to his behaviors. However, when he was transferred to the Veterans Affairs Community Living Center and received care primarily from the Behavioral Recovery Outreach Team, seizure mats were used so he could continue "working." The interprofessional team also introduced a consistent bowel regimen, determined the best ways and times to engage with him, and did not challenge his perception of reality. These interventions reduced aggression and the use of restraints, and he appeared more content.

## Discussion

The exact pathogenesis of BPSD is not fully understood; however, the cholinergic hypothesis postulates that cholinergic loss may be the cause of attention deficits leading to psychosis, apathy, and agitation in patients with dementia [5]. Evidence also points to alterations in serotonergic receptors as playing a role in nearly all BPSD, including affective symptoms, hyperactivity, irritation, disinhibition, aggression, apathy, and psychosis [2]. Finally, dopaminergic activation can contribute to the psychotic features of BPSD, while decreased dopamine signaling likely contributes to apathy [2]. Pharmacologic therapies targeting these pathways, particularly antipsychotics and acetylcholinesterase inhibitors, have shown modest benefits [6]. However, non-pharmacologic interventions have shown similar effects with far fewer adverse events, emphasizing the importance of these methods as first-line therapy [6]. If pharmacologic therapy is necessary, treatment should be individualized with clear symptom targets.

In our patient, psychotropics were periodically adjusted while the main focus was on non-pharmacologic interventions. Staff only used psychotropics for behavioral issues that caused distress or created safety concerns for the patient or staff. For example, when he was upset or yelling at his “workers,” psychotropics were used to decrease distress. However, his loud singing was not a sign of distress, so no medication changes were made. This approach, though patient-centered, required significant buy-in from staff since non-pharmacologic interventions often require more time and effort. Thankfully, the staff caring for this patient were flexible in taking extra time to ensure safety while still allowing for meaningful expression through his “tiling work.” One of the nursing staff members who knows him well prioritized educating other nurses about the best ways to engage with him, his preferences, and what triggers to avoid. This continuity of care can aid in promoting the "continuation of self and normality," a core principle of person-centered care [7]. Additionally, the implementation of person-centered care has been shown to reduce BPSD [8].

Perineal care and incontinence were major triggers for verbal and physical aggression. By incorporating nursing staff suggestions for a consistent bowel program with morning suppositories, our patient was able to sleep or “work” afterward with fewer interruptions. It also reduced the frequency of bowel incontinence, which can be very challenging to clean up. If he was “working” on the floor and in need of care, staff would tell him it is “break time.” If he became aggressive with any aspect of care that could be safely delayed, staff were encouraged to step back and give him some time to “cool down” and try again later. Though some aspects of this approach are not novel, the way staff framed the care they needed to provide in the context of his "work" is unique. For other patients, staff should be encouraged to assess and accommodate the patient's current perception of reality into the care they provide. Additionally, some interventions that can be more easily generalized, like doll therapy, animal-assisted and pet-robot interventions, and music therapy, have been shown to significantly reduce BPSD [9,10,11]. All of the approaches mentioned thus far focus on a key aspect of quality dementia care: meeting the patient in their reality to minimize distress and maximize the quality of life.

Some patients with dementia may also exhibit sexual disinhibition, which is often difficult to manage effectively. Since several medications have been shown to worsen sexual disinhibition, a thorough medication review along with a full medical, social, and sexual history is important [12]. Additionally, the review by Sarangi et al. summarizes treatments and their effectiveness in different types of dementia [12]. Just like BPSD, sexual disinhibition requires person-centered care and a supportive interprofessional team to provide the best care for these patients and support staff members through challenging situations.

Individualized interventions, such as the incorporation of our patients' seizure mats, were the most important aspect of person-centered dementia care. While maintaining the safety of the patient and staff must always be a priority, some interventions increase adverse events, decrease quality of life, and should be avoided if possible. For example, physical restraints are associated with an increased risk of death, falls, serious injury, and longer hospital stays [13]. Restraints are often used when providers feel there is no other option for controlling behavior [13]. Our interprofessional team includes a physical therapist, an occupational therapist, a social worker, a psychologist, nurses, and a physician. Through the involvement of this collaborative team, staff directly working with the patient feel more supported, behaviors are more easily anticipated and avoided, and restraints have not been required for over two years.

The use of antipsychotics in older adults with dementia also carries an increased mortality risk [14]. Our patient is on regularly scheduled doses of quetiapine and lorazepam. This was initiated to reduce his distress, particularly his agitation with care, and gradual dose reductions are performed periodically to ensure that he is on the lowest effective antipsychotic and benzodiazepine doses. This approach discourages the overuse of medications, particularly sedatives, and encourages a constant re-evaluation of medication regimens to find the minimum necessary. A summary of successful and discouraged behavioral interventions for our patient is summarized in Table 1.

**Table 1:** Examples of person-centered BPSD management strategies BPSD: Behavioral and psychological symptoms of dementia

Successful behavioral interventions	Discouraged behavioral interventions
Interprofessional communication	Restraints [13]
Identify triggers and signs of distress [4]	Over-sedation with psychotropics [14]
Implement interventions that ensure safety while allowing for meaningful expression [7]	Attempting to challenge or correct a patient’s logic or perception of reality
Engage patient’s current perception of the world, which is often related to key aspects of their former identity and roles	

Finally, the patient’s family and care team chose to pursue a hospice-like approach despite not meeting hospice eligibility criteria. Many patients with significant behaviors secondary to dementia do not meet hospice eligibility criteria because they are not bedridden [15]. However, this approach allowed for further reduction in behavior triggers by reducing medical care that was not symptom-based.

## Conclusions

In summary, many of the interventions used for behavioral management in our patient were focused on engaging the patient’s current perception of the world, often about his identity working in flooring, and redirecting by it. Thankfully, this patient's perception of reality was reasonably accommodated using seizure mats. If, for example, he had believed he was still in the military or working in maintenance, his reality would have been more challenging to accommodate. Staff would have to think creatively about ways to safely accommodate these realities, perhaps by using play tools or addressing the patient by their military title. Restraints, which were used during initial acute hospitalization, have not been required for over two years, and the use of psychotropics has been minimized as much as possible. A little over two years after his admission to the Veterans Affairs, he continues his “tiling work.” At times he has angry outbursts at his “workers,” but the staff is more equipped and able to anticipate these outbursts and respond appropriately. If not working, he is usually lying or sleeping in bed. His care team continues a comfort-based hospice approach with the goal of reducing distress and maintaining a good quality of life.
